# Dietary Total Antioxidant Capacity—A New Indicator of Healthy Diet Quality in Cardiovascular Diseases: A Polish Cross-Sectional Study

**DOI:** 10.3390/nu14153219

**Published:** 2022-08-06

**Authors:** Małgorzata Elżbieta Zujko, Anna Waśkiewicz, Anna Maria Witkowska, Alicja Cicha-Mikołajczyk, Kinga Zujko, Wojciech Drygas

**Affiliations:** 1 Department of Food Biotechnology, Faculty of Health Sciences, Medical University of Białystok, Szpitalna 37, 15-295 Białystok, Poland; 2 Department of Epidemiology, Cardiovascular Disease Prevention and Health Promotion, National Institute of Cardiology, Alpejska 42, 04-628 Warsaw, Poland; 3 Department of Cardiology, Medical University of Białystok, M. Skłodowskiej-Curie 24a, 15-276 Białystok, Poland; 4 Department of Social and Preventive Medicine, Faculty of Health Sciences, Medical University of Łódź, Hallera 1, 90-001 Łódź, Poland

**Keywords:** cardiovascular disease, dietary total antioxidant capacity, FRAP, population

## Abstract

This study aimed to assess the relationship between the dietary total antioxidant capacity (DTAC) and the occurrence of cardiovascular diseases (CVDs), as well as healthy diet quality, in a representative sample (*n* = 5690) of the whole Polish adult population (WOBASZ II study). Daily food consumption was estimated by the single 24 h dietary recall method. Antioxidant vitamins (C, E, and β-carotene) and minerals (Zn, Fe, Mn, and Cu) from the diet and supplements were calculated using 5D Diet software, and dietary total polyphenol intake (DTPI) was determined using the Phenol-Explorer database and our database. Total diet quality was measured by the Healthy Diet Indicator (HDI) based on World Health Organization (WHO) recommendations for the prevention of CVD. DTAC was calculated using the data on food consumption and the antioxidant potential of foods measured by the FRAP (ferric ion reducing antioxidant potential) method. It was shown that higher DTAC was associated with a higher intake of polyphenols, antioxidant vitamins, and minerals. Moreover, a higher quartile of DTAC was associated with a reduced odds ratio for cardiovascular diseases in a Polish population, as well as with a higher HDI. Therefore, dietary recommendations for the prevention and therapy of CVDs should take into account a high DTAC. DTAC, measured by the FRAP method, can be considered an indicator of healthy diet quality.

## 1. Introduction

According to the World Health Organization (WHO) report, cardiovascular diseases (CVDs) are the leading cause of death worldwide. It is estimated that in 2019, deaths from CVDs accounted for 32% of all deaths. Most cardiovascular diseases can be prevented by changing modifiable risk factors, such as unhealthy diet, obesity, tobacco use, physical inactivity, and extensive alcohol consumption [1].

All of the recommended dietary patterns for CVD prevention, including the Mediterranean diet and the Dietary Approaches to Stop Hypertension model, emphasize the importance of high diet quality. A common feature of such dietary patterns is the high consumption of healthy foods, i.e., vegetables, fruits, whole grains, legumes, nuts and seeds, low-fat dairy, fish, and unprocessed lean meats and poultry, and low consumption of energy-dense, nutrient-poor foods rich in saturated fat, trans fat, added sugar and salt [2,3].

Epidemiological research has globally demonstrated that plant-based foods, which contain polyphenols, vitamins, minerals, and fiber, with proven antioxidant activity, can prevent CVDs [4,5]. Our previous study showed that individual diet modifications in terms of a higher intake of polyphenols (flavonoids and anthocyanins), fiber, and polyunsaturated fatty acids (PUFA) and a lower intake of saturated fatty acids (SFA) had a significant impact on the improvement of some metabolic syndrome risk factors, such as waist circumference, fasting glucose, and HDL cholesterol [6]. However, no association was found between lignan intake and the prevalence of CVDs [7].

A new approach to a healthy diet is the assessment of dietary total antioxidant capacity (DTAC). The whole diet contains various antioxidants (vitamins: C, E, and carotenoids; minerals: Zn, Fe, Mn, Cu, and Se; polyphenols) with additional or synergistic effects. Several assays are available to measure antioxidants in foods, but the largest database is based on the FRAP (ferric ion reducing antioxidant potential) method [8]. The current FRAP database includes more than 3100 different foods, beverages, spices, herbs, and supplements purchased at local stores and markets around the world [9]. Some authors suggest that dietary FRAP may be considered an appropriate measure of dietary quality because it positively correlates with well-known indicators of a healthy diet [10].

The epidemiological evidence of a relationship between DTAC measured by the FRAP method and CVDs is limited. Findings from a meta-analysis of prospective cohort studies revealed that higher dietary FRAP was associated with a lower risk of CVD mortality [11]. In some studies, DTAC was inversely associated with cardiovascular events and cardiometabolic risk factors [12], blood pressure and diabetes [13], prediabetes and insulin resistance [14,15], and lipid biomarkers [15]. In the other study, no association was found between DTAC and waist circumference, glucose level, insulin resistance, or lipid biomarkers. However, the study was conducted on a small group of patients with nonalcoholic fatty liver disease [16].

This study aimed to assess the relationship between the dietary total antioxidant capacity and the occurrence of cardiovascular diseases, as well as healthy diet quality, in the Polish adult population. This cross-sectional study supplements the existing knowledge in this field.

## 2. Materials and Methods

### 2.1. Ethical Approval

The study was conducted in accordance with the Helsinki Declaration and Good Clinical Practice. Approval for the WOBASZ II study was obtained from the Bioethics Committee at the National Institute of Cardiology (No. 1344), which also approved the current study (No. 1837). Written informed consent was obtained from all participants.

### 2.2. Study Group

The subjects (5690) were participants of the National Multicenter Health Survey II (WOBASZ II) conducted by the National Institute of Cardiology in Warsaw in 2013–2014. It was a cross-sectional study aimed at investigating the determinants of chronic non-communicable diseases in a representative sample of Polish adults aged 20 years and older. The design and methods of the WOBASZ II study have been described in detail elsewhere [17].

Baseline information about participants (smoking, educational level, physical activity, and diseases) was collected using a standardized questionnaire developed for the WOBASZ II study. Physical activity was classified as follows: low level—physical activity for at least 30 min a day once a week or less; middle level—physical activity for at least 30 min a day 2–3 times a week; and high level—physical activity for at least 30 min a day ≥ 4 times a week.

Subjects were classified as having CVD if they had: coronary heart disease, myocardial infarction, stroke, atrial fibrillation and/or other cardiac arrhythmias, peripheral vascular disease of the lower limbs, heart failure, coronary angioplasty or coronary artery bypass grafting, and implanted pacemaker or cardioverter-defibrillator, as was reported previously [18].

### 2.3. Clinical Measurements

The measurements of body mass, height, and waist circumference were performed by trained nurses using standardized procedures. Body mass index (BMI) was calculated as body mass in kilograms divided by squared height in meters, and a BMI of 18.5–24.9 kg/m^2^ was defined as normal body mass [19]. Blood pressure (BP) was measured three times on the right arm after 5 min of rest in a sitting position at 1 min intervals using automatic devices (AND UA-631), and the final BP was reported as the mean of the second and third measurements. Hypertension was diagnosed when systolic blood pressure (BPs) ≥ 140 mm Hg and/or diastolic blood pressure (BPd) ≥ 90 mm Hg and/or when antihypertensive drugs were used [20]. Biochemical analyses were performed at the Diagnostyka Central Laboratory of the National Institute of Cardiology in Warsaw. Diabetes was diagnosed when the fasting glucose (FG) level was ≥126 mg/dL or participants were taking antidiabetic drugs [21]. Hypercholesterolemia was defined when fasting total cholesterol (TC) was ≥190 mg/dL or low-density lipoprotein cholesterol (LDL-C) was ≥115 mg/dL or subjects were taking lipid-lowering medication, and hypertriglyceridemia was defined when the fasting triglyceride level (TG) was ≥150 mg/dL or participants used lipid-lowering drugs [20].

A diagnosis of metabolic syndrome (MetS) was made when at least three of five risk factors were identified: waist circumference (≥94 cm for men and ≥80 cm for women), TG (≥150 mg/dL), HDL-C (<40 mg/dL for men and <50 for women), BPs ≥ 130 mm Hg and/or BPd ≥ 85 mm Hg), and FG (≥100 mg/dL) [22].

### 2.4. Dietary Assessment

Dietary and supplement intake assessment was performed by trained interviewers using a single 24 h dietary recall method. Food portion sizes were estimated using an Album of Photographs of Food Products and Dishes [23]. Subjects were asked if they had taken any form of a dietary supplement on the recall day, and the supplement type, name brand, and dose were recorded. Nutrient intake (including vitamins and minerals) from the diet was calculated based on the amount of food consumed with the use of Polish Food Composition Tables [24].

### 2.5. Assessment of Dietary Antioxidants

Antioxidant vitamin (C, E, and β-carotene) and mineral (Zn, Fe, Mn, and Cu) intake from both food and dietary supplements were taken into account. In the case of vitamins, losses during technological processes and food preparation were deducted. The amounts of vitamins and minerals from diet and supplements were estimated using the NFNI (National Food and Nutrition Institute) 5D Diet software (IŻŻ Diet 5D).

Dietary total polyphenol intake (DTPI) was estimated using the online Phenol-Explorer database [25] and our database [26,27].

Dietary total antioxidant capacity (DTAC) was estimated using the FRAP method published by Carlsen [28] and our database [26,27].

### 2.6. Assessment of Healthy Diet Indicator (HDI)

Total diet quality was measured using the Healthy Diet Indicator (HDI) based on World Health Organization (WHO) recommendations for the prevention of CVDs [29] and described in [30]. The HDI includes the consumption of six nutrients (saturated fatty acids, polyunsaturated fatty acids, cholesterol, protein, fiber, and free sugars) and the sum of fruits and vegetables within the recommended range, with 1 point awarded for the recommended intake of a given nutrient, and 0 points if intake was not consistent with guidelines. The maximum score for an optimal diet was 7 points.

In the present study, an HDI score equal to at least 5 points was arbitrarily assumed as an indicator of a proper diet.

### 2.7. Statistical Analysis

The study participants were divided into four subgroups according to the quartile distribution of DTAC for men and women separately and for the total group.

Quantitative variables are presented as the mean (standard deviation) and/or median (interquartile range), while qualitative variables are reported as the number and percentage. The Wilcoxon test and the chi-square test were used for comparisons between CVD and non-CVD subgroups, if appropriate.

Mean values of antioxidants and/or polyphenol intake from food and supplements, as well as the HDI score, with a 95% confidence interval (95% CI) were calculated by a general linear model with the Tukey–Kramer adjustment for multiple comparisons. The Cochran–Armitage test for trend was applied to assess for an association between the prevalence of a proper diet (HDI ≥ 5) and DTAC over quartiles.

Logistic regression models were used to assess the relationship between DTAC and the prevalence of CVDs. Three models were applied: model 1: crude in men and women or adjusted for sex in the total group; model 2: adjusted for age, BMI, HDI, smoking status, and alcohol intake in men and women and additionally for sex in the total group; model 3: adjusted for age, BMI, HDI, smoking status, alcohol intake, educational level, and physical activity in men and women and additionally for sex in the total group. The first quartile (Q1) in each model was adopted as a reference. The results of logistic regression analysis are presented as odds ratios (ORs) and 95% confidence intervals.

All statistical analyses were performed using SAS software version 9.4 (SAS Institute Inc., Cary, NC, USA). A p-value less than 0.05 was considered statistically significant.

## 3. Results

Baseline characteristics of the study population according to CVDs for both sexes are presented in Table 1. Among 5690 analyzed participants (2554 men and 3136 women), 1138 (20%) had CVD (494 men and 644 women). It was shown that CVD participants were older (respectively: 62.3 ± 14.1 years vs. 46.4 ± 15.4 years), had higher BMI (respectively: 28.8 ± 5.1 kg/m^2^ vs. 26.8 ± 5.1 kg/m^2^), consumed less fiber (respectively: 18.3 ± 8.2 g/day vs. 19.3 ± 8.7 g/day), and less frequently had a higher educational level (respectively: 11.52% vs. 21.93%). On the other hand, people with CVDs were less likely to smoke cigarettes (respectively: 16.9% vs. 24.87%) and consumed less energy (respectively: 1755 ± 729 kcal vs. 2017 ± 867 kcal) and alcohol (respectively: 1.5 ± 10.2 g/day vs. 2.7 ± 15.0 g/day). Over 50% of participants had a low level of physical activity. The recommended moderate physical activity was more common in non-CVD in comparison to CVD patients (16.14% vs. 11.89%). Moreover, CVD compared to non-CVD participants more often had hypertension (68.7% vs. 39.3%), diabetes mellitus (22.84% vs. 7.81%), hypercholesterolemia (79.33% vs. 66.25%), hypertriglyceridemia (32.17% vs. 27.39%), and metabolic syndrome (52.2% vs. 31.5%).

Overall, CVD participants were older and less educated than those without CVD, which could have resulted in poorer lifestyles (food choices and exercise) and the development of obesity, diabetes, hypertension, and dyslipidemia. Health-promoting behaviors of people with CVDs were limited to smoking and drinking less alcohol and consuming less energy compared to people without CVDs. Therefore, further analyses were adjusted for confounding factors.

A comparison of antioxidant intake from food and supplements between CVD and non-CVD participants is shown in Table 2. The results were adjusted for age in men and women and for sex and age in the total group. It was detected that the diet of non-CVD subjects contained significantly more DTAC, DTPI, Fe, and Cu in comparison to CVD subjects for men and the total group and more Zn compared to the total group. However, after recalculation of the results per 1000 kcal of the diet, DTAC was significantly higher only in non-CVD relative to CVD men. Moreover, consumption of β-carotene and Mn per 1000 kcal of the diet was significantly lower in non-CVD than in CVD men and the total group. In women, no significant differences were found between CVD and non-CVD participants.

Antioxidant intake from food and supplements by quartiles of DTAC in men, in women, and in the total group is listed in Table 3. The results were adjusted for age in men and women and for sex and age in the total group. It was shown that higher DTAC was associated with higher DTPI, intake of antioxidant vitamins (C, E, and β-carotene), and minerals (Zn, Fe, Mn, and Cu) in men, women, and the total group. After recalculation of the results per 1000 kcal of the diet, significant positive associations were found only for DTPI, vitamin C, and Cu in men and women and for DTPI, vitamin C, Fe, Mn, and Cu in the total group. However, inverse associations were found for Zn per 1000 kcal of diet in men and women and β-carotene and Zn per 1000 kcal of the diet in the total group.

The prevalence and OR (95% CI) of CVDs by quartiles of DTAC are shown in Table 4. Among men, the mean percentage of CVD prevalence was 23.1% in the first quartile (Q1), 21.8% in the second quartile (Q2), 17.2% in the third quartile (Q3), and 15.2% in the fourth quartile (Q4). Among women, it was: Q1—23.7%; Q2—21.1%; Q3—18.3%; and Q4—19.0%. In the total group, the prevalence of CVD was: Q1—23.3%; Q2—21.4%; Q3—18.1%; and Q4—17.2%. The prevalence of CVDs decreased significantly with increasing DTAC.

When the analysis was adjusted for multiple variables (model 3), the OR of CVDs in men was 30.2% lower in Q3 (OR = 0.698, 95% CI = 0.504–0.967, *p* = 0.0306) and 39.0% lower in Q4 (OR = 0.610, 95% CI = 0.436–0.855, *p* = 0.0041) in comparison to Q1. In women, the OR of CVDs was 27.4% lower in Q3 (OR = 0.726, 95% CI = 0.550–0.958, *p* = 0.0237) relative to Q1. In the total group, the OR of CVDs was 27.4% lower in Q3 (OR = 0.726, 95% CI = 0.588–0.895, *p* = 0.0028) and 24.8% lower in Q4 (OR = 0.752, 95% CI = 0.605–0.935, *p* = 0.0102) in comparison to Q1.

**Figure 1:**
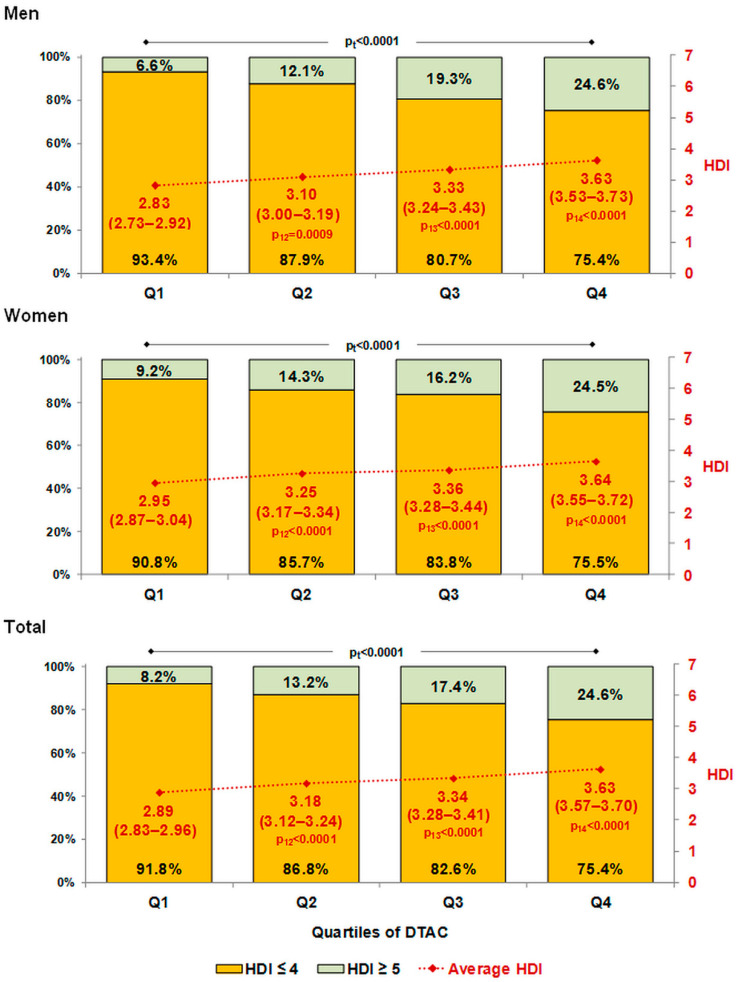
Distribution of HDI score (HDI ≥ 5 vs. HDI ≤ 4) and average HDI score (95% CI) by quartiles of DTAC. Mean HDI adjusted for age in men and women and additionally for sex in the total group. p_t_—p-value for trend test; p_12_, p_13_, and p_14_—p-values for comparisons with reference quartile Q1. DTAC—dietary total antioxidant capacity; HDI—Healthy Diet Indicator. Difference between average HDI was tested using a general linear model with Tukey–Kramer adjustment for multiple comparisons, while the distribution of HDI was tested by Cochran–Armitage test for trend.

The mean values of HDI, after adjustment for age in men and women and additionally for sex in the total group, are presented in Figure 1. The average HDI increased significantly across quartiles of DTAC from 2.83 (95% CI = 2.72–2.92) to 3.63 (95% CI = 3.53–3.73) in men, from 2.95 (95% CI = 2.87–3.04) to 3.64 (95% CI = 3.55–3.72) in women, and from 2.89 (95% CI = 2.83–2.96) to 3.63 (95% CI = 3.57–3.70) overall.

Furthermore, the percentage of people who followed a healthy diet was also associated with an increase in DTAC. In our study, a proper diet (HDI ≥ 5) was followed by 6.6% and 24.6% of men, 9.2% and 24.5% of women, and 8.2% and 24.6% of the total in the first (Q1) and fourth (Q4) quartiles of DTAC, respectively (Figure 1). 

## 4. Discussion

In the present study, we evaluated for the first time the relationship between DTAC, measured by the FRAP method, and the prevalence of CVDs in a representative sample of the Polish adult population (WOBASZ II study). It was found that higher DTAC was associated with a lower prevalence of CVDs. The odds ratio of developing CVD, after adjustment for confounding variables (model 3), was 24.8–39.0% lower in the third and fourth quartiles in comparison to the first quartile of DTAC.

CVDs are the leading cause of death and disability around the world [1]. While genetic and environmental contributors to developing CVD are important, modifiable risk factors associated with lifestyle also play a large role. Food choices influence the development of obesity, hypertension, and dyslipidemia, which increase the risk of CVD [31]. Therefore, guidelines for the prevention of CVDs also include healthy diet strategies [20]. In our study, people with CVDs were more likely to have obesity, hypertension, diabetes mellitus, hypercholesterolemia, hypertriglyceridemia, and metabolic syndrome.

A large body of evidence supports the intake of natural nutrients such as polyphenols [32] and antioxidant vitamins [33], as well as dietary patterns such as the Mediterranean diet [34], Dietary Approaches to Stop Hypertension [35], the Nordic Diet [36], and Traditional Asian Diets [37,38,39], can prevent CVDs. The findings of three large prospective cohorts with up to 32 years of follow-up showed that greater adherence to various healthy eating patterns was associated with a lower risk of CVD [40]. Healthy dietary patterns share common characteristics based on higher consumption of vegetables, fruits, legumes, nuts and seeds, and whole grains, which are the most important sources of antioxidants [41]. In contrast to natural antioxidants, exogenous supplementation of antioxidant vitamins and minerals in people without nutritional deficiencies has no beneficial effects on the prevention of CVDs [42]. The findings of the NHANES study showed that US adults with high DTAC showed higher adherence to popular diet quality index scores: HEI-2015 (Healthy Eating Index-2015), AHEI-2010 (Alternative Healthy Eating Index-2010), aMED (alternate Mediterranean Diet), and DASH (Dietary Approaches to Stop Hypertension). However, in this study, dietary total antioxidant capacity was measured using the ABTS (2,2′-azino-bis-3-ethylbenzthiazoline-6-sulphonic acid) assay in individual dietary antioxidant vitamins and polyphenols and expressed as the sum of individual antioxidant capacities [43]. In our study, DTAC measured by the FRAP method was positively associated with the intake of individual dietary antioxidants (from foods and supplements), such as total polyphenols, antioxidant vitamins (C, E, and β-carotene), and minerals (Fe, Zn, Cu, and Mn (as antioxidant enzyme cofactors)). However, after recalculation of the results per 1000 kcal of the diet, significant positive associations were found only for DTPI, vitamin C, and Cu in men and women and for DTPI, vitamin C, Fe, Mn, and Cu in the total group. Surprisingly, inverse associations were found for Zn in men and women and for β-carotene and Zn in the total group. The measurement of the total antioxidant potential of the diet includes different individual antioxidants with synergistic, additive, or inverse effects. The consumption of individual antioxidants was measured in both the diet and supplements, while excessive consumption of antioxidants with supplements may have a pro-oxidative effect.

In this study, DTAC was positively associated with healthy diet quality measured by the HDI score, although the quality of the diet in the Polish adult population was relatively low (mean HDI less than 4 in men, women, and total).

Dietary antioxidants can support the action of endogenous antioxidants (enzymatic, e.g., superoxide dismutase, glutathione peroxidase, and catalase, and non-enzymatic, e.g., glutathione, metal-binding proteins, uric acid, melatonin, and bilirubin) in alleviating the destructive effects of oxidative stress, e.g., can inhibit LDL-C oxidation and prevent endothelial dysfunction. The overproduction of free radicals (ROS—reactive oxygen species; RNS—reactive nitrogen species) in oxidative stress can damage the body’s DNA, as well as proteins and lipids, and lead to non-communicable diseases, including CVDs. There is emerging evidence that inflammatory mechanisms and oxidative stress lead to atherosclerosis, arterial hypertension, arterial fibrillation, and heart failure. However, increasing oxidative stress can be considered a contributing factor, not the primary pathophysiologic mechanism, because CVDs are very complex in their pathogenesis [44].

In previous population studies, DTAC, measured by different methods, was inversely associated with prediabetes, insulin resistance, and diabetes [14,45,46], cancer [47], myocardial infarction [48], heart failure [49], and stroke [50]. In a large cross-sectional study, DTAC, measured by the FRAP method, was inversely associated with the hypertriglyceridemic waist phenotype and amputation due to arterial disease in people undergoing secondary care for CVDs [12]. In another cross-sectional study, high DTAC, measured by the FRAP method, had a protective effect against oxidative DNA damage in individuals at cardiovascular risk [51]. Findings from a meta-analysis of prospective cohort studies have shown that high DTAC was associated with a lower risk of mortality from all causes, cancer, and CVDs. In addition, a 5 mmol/day increase in DTAC, based on the FRAP method, was associated with a 7% lower risk of all-cause mortality [11].

Population studies on the relationship between DTAC, measured by the FRAP method, and CVDs are quite limited. Therefore, further research is needed to investigate these associations and mechanisms of action.

### Limitations

The present study has some strengths and limitations. The strength of this study is that it was a cross-sectional study conducted on a large, representative sample of the adult Polish population. Moreover, dietary antioxidants were calculated from foods and supplements. The first limitation of this study is the use of a single 24 h recall, which does not take into account habitual food intake, although this method is commonly used in large population studies [43]. Moreover, participants who indicated that their 24 h recall differed from their typical diet (usual eating habits, typical of most days of the year) were excluded from the study. The second limitation is that the FRAP database, although very extensive, did not contain all foods, beverages, herbs, and supplements. Thirdly, this study did not take into account the consumption of selenium, which is also a component of antioxidant enzymes, because Polish Food Composition Tables do not contain this micronutrient.

## 5. Conclusions

This study demonstrated that a higher quartile of DTAC was significantly associated with a reduced odds ratio for cardiovascular diseases in the Polish adult population, as well as with a higher Healthy Diet Indicator. Therefore, dietary recommendations for the prevention and therapy of cardiovascular diseases should take into account a high DTAC. DTAC, measured by the FRAP method, can be considered an indicator of healthy diet quality.

## Figures and Tables

**Table 1:** Baseline characteristics of the study population according to CVDs for both sexes and overall.

Variables	Men (*n* = 2554)		*p*	Women (*n* = 3136)		*p*	Total (*n* = 5690)		*p*
	CVD	Non-CVD		CVD	Non-CVD		CVD	Non-CVD	
	*n* = 494	*n* = 2060		*n* = 644	*n* = 2492		*n* = 1138	*n* = 4552	
Age [years], Mean ± SD	62.4 ± 13.2	45.5 ± 15.2	<0.0001	62.3 ± 14.8	47.1 ± 15.5	<0.0001	62.3 ± 14.1	46.4 ± 15.4	<0.0001
BMI [kg/m^2^], Mean ± SD	28.5 ± 4.7	27.2 ± 4.5	<0.0001	29.0 ± 5.3	26.4 ± 5.6	<0.0001	28.8 ± 5.1	26.8 ± 5.1	<0.0001
Current smoking, *n* (%)	93 (18.90)	645 (31.36)	<0.0001	99 (15.37)	486 (19.51)	0.0163	192 (16.90)	1131 (24.87)	<0.0001
Educational level, *n* (%)									
Under middle	118 (23.94)	258 (12.54)	<0.0001	221 (34.32)	376 (15.11)	<0.0001	339 (29.82)	634 (13.95)	<0.0001
Vocational	156 (31.64)	642 (31.20)		110 (17.08)	464 (18.65)		266 (23.39)	1106 (24.33)	
Middle	166 (33.67)	775 (37.66)		235 (36.49)	1034 (41.56)		401 (35.27)	1809 (39.79)	
Higher	53 (10.75)	383 (18.61)		78 (12.11)	614 (24.68)		131 (11.52)	997 (21.93)	
Physical activity, *n* (%)									
Low level	257 (52.34)	1142 (55.60)	0.0039	363 (56.37)	1314 (52.96)	0.0403	620 (54.63)	2456 (54.16)	0.0017
Middle level	57 (11.61)	320 (15.58)		78 (12.11)	412 (16.61)		135 (11.89)	732 (16.14)	
High level	165 (33.60)	535 (26.05)		187 (29.04)	705 (28.42)		352 (31.01)	1240 (27.34)	
Seasonally	12 (2.44)	57 (2.78)		16 (2.48)	50 (2.02)		28 (2.47)	107 (2.36)	
Energy [kcal/day], Mean ± SD	2034 ± 829	2385 ± 947	<0.0001	1542 ± 555	1713 ± 654	<0.0001	1755 ± 729	2017 ± 867	<0.0001
Dietary fiber [g/day], Mean ± SD	20.3 ± 8.7	21.1 ± 9.2	0.0733	16.8 ± 7.4	17.7 ± 7.8	0.0019	18.3 ± 8.2	19.3 ± 8.7	0.0005
Alcohol intake [g/day], Mean ± SD	3.1 ± 15.1	5.0 ± 21.0	0.0111	0.3 ± 2.7	0.7 ± 6.4	0.0792	1.5 ± 10.2	2.7 ± 15.0	0.0014
Hypertension, *n* (%)	359 (73.27)	890 (43.84)	<0.0001	418 (65.21)	874 (35.56)	<0.0001	777 (68.70)	1764 (39.30)	<0.0001
Diabetes mellitus, *n* (%)	129 (27.22)	161 (8.17)	<0.0001	119 (19.44)	178 (7.51)	<0.0001	248 (22.84)	339 (7.81)	<0.0001
Hypercholesterolemia, *n* (%)	401 (82.68)	1376 (68.97)	<0.0001	493 (76.79)	1563 (64.03)	<0.0001	894 (79.33)	2939 (66.25)	<0.0001
Hypertriglyceridemia, *n* (%)	181 (38.19)	699 (35.50)	0.2742	168 (27.50)	494 (20.70)	0.0003	349 (32.17)	1193 (27.39)	0.0018
Metabolic syndrome, *n* (%)	272 (55.06)	728 (35.34)	<0.0001	322 (50.00)	706 (28.33)	<0.0001	594 (52.20)	1434 (31.50)	<0.0001

CVD—cardiovascular disease; SD—standard deviation; *n*—number. Differences between quantitative and qualitative variables were tested by Wilcoxon rank sum test or Chi2 test, respectively.

**Table 2:** Comparison of antioxidant intake from food and supplements between CVD and non-CVD participants according to sex and overall.

Variables	Men ^1^		*p*	Women ^1^		*p*	Total ^2^		*p*
	CVD	Non-CVD		CVD	Non-CVD		CVD	Non-CVD	
	Mean (95% CI)			Mean (95% CI)			Mean (95% CI)		
DTAC [mmol/d]	11.06 (10.35–11.77)	12.69 (12.36–13.02)	<0.0001	12.15 (11.55–12.74)	12.29 (12.00–12.58)	0.6776	11.70 (11.24–12.15)	12.47 (12.25–12.69)	0.0033
DTAC/1000 kcal	5.42 (5.09–5.75)	5.81 (5.66–5.97)	0.0388	8.18 (7.77–8.59)	7.78 (7.58–7.98)	0.0931	6.86 (6.59–7.13)	6.79 (6.66–6.92)	0.6511
DTPI [mg/d]	1948 (1858–2037)	2101 (2060–2143)	0.0029	1935 (1866–2004)	2000 (1967–2034)	0.1046	1947 (1892–2002)	2050 (2023–2076)	0.0013
DTPI/1000 kcal	945 (905–986)	954 (936–973)	0.7086	1298 (1249–1347)	1255 (1232–1279)	0.1302	1126 (1094–1159)	1104 (1088–1120)	0.2346
Vitamin C [mg/d]	85.9 (78.2–93.7)	87.9 (84.3–91.5)	0.6611	92.2 (84.1–100.3)	96.1 (92.2–100.1)	0.4020	88.9 (83.2–94.6)	92.0 (89.3–94.7)	0.3437
Vitamin C/1000 kcal	43.5 (39.5–47.4)	40.5 (38.6–42.3)	0.1851	63.5 (57.5–69.4)	61.2 (58.3–64.1)	0.5097	53.5 (49.7–57.3)	50.8 (49.0–52.6)	0.2218
Vitamin E [mg/d]	12.38 (11.60–13.16)	12.67 (12.30–13.03)	0.5262	10.47 (9.28–11.65)	11.11 (10.54–11.69)	0.3495	11.38 (10.63–12.13)	11.90 (11.54–12.25)	0.2366
Vitamin E/1000 kcal	5.71 (5.38–6.04)	5.52 (5.36–5.67)	0.3043	6.82 (5.90–7.74)	6.86 (6.41–7.31)	0.9438	6.25 (5.71–6.79)	6.19 (5.93–6.45)	0.8444
β-Carotene [μg/d]	2980 (2685–3275)	2972 (2836–3109)	0.9652	3210 (2936–3484)	2923 (2789–3056)	0.0716	3109 (2908–3310)	2944 (2848–3041)	0.1577
β-Carotene/1000 kcal	1601 (1443–1759)	1396 (1322–1469)	0.0246	2135 (1933–2337)	1927 (1829–2025)	0.0764	1870 (1737–2003)	1661 (1597–1725)	0.0067
Zinc [mg/d]	11.34 (10.86–11.82)	11.62 (11.40–11.85)	0.3089	8.26 (7.91–8.61)	8.64 (8.47–8.81)	0.0570	9.78 (9.49–10.07)	10.13 (10.00–10.27)	0.0326
Zinc/1000 kcal	5.28 (5.12–5.44)	5.10 (5.03–5.18)	0.0506	5.39 (5.19–5.59)	5.29 (5.19–5.39)	0.4099	5.33 (5.20–5.46)	5.20 (5.13–5.26)	0.0809
Iron [mg/d]	11.99 (11.40–12.57)	12.70 (12.43–12.97)	0.0348	10.05 (9.38–10.71)	10.41 (10.09–10.73)	0.3369	11.03 (10.58–11.48)	11.55 (11.33–11.77)	0.0480
Iron/1000 kcal	5.61 (5.40–5.82)	5.59 (5.49–5.69)	0.8788	6.55 (6.16–6.93)	6.35 (6.16–6.53)	0.3675	6.10 (5.86–6.33)	5.96 (5.85–6.08)	0.3302
Manganese [mg/d]	4.75 (4.53–4.96)	4.66 (4.56–4.76)	0.4996	3.90 (3.74–4.06)	4.06 (3.98–4.14)	0.0842	4.31 (4.18–4.44)	4.36 (4.30–4.43)	0.4498
Manganese/1000 kcal	2.31 (2.21–2.41)	2.14 (2.09–2.18)	0.0032	2.65 (2.54–2.76)	2.56 (2.51–2.62)	0.1539	2.48 (2.40–2.55)	2.35 (2.32–2.39)	0.0038
Copper [mg/d]	1.21 (1.15–1.26)	1.27 (1.24–1.29)	0.0429	1.03 (0.99–1.07)	1.07 (1.05–1.09)	0.0873	1.12 (1.08–1.15)	1.17 (1.15–1.18)	0.0076
Copper/1000 kcal	0.568 (0.549–0.587)	0.562 (0.553–0.571)	0.5883	0.675 (0.650–0.700)	0.656 (0.644–0.668)	0.1971	0.623 (0.606–0.639)	0.609 (0.601–0.617)	0.1450

^1^—Adjusted for age; ^2^—adjusted for sex and age; DTAC—dietary total antioxidant capacity; DTPI—dietary total polyphenol intake; CVD—cardiovascular disease; CI—confidence interval. The general linear model was applied to identify differences in all variables between adjusted means of CVD and non-CVD subgroups.

**Table 3:** Antioxidant intake from food and supplements by quartiles of DTAC according to sex and overall.

Variables	Men				*p*	Women				*p*	Total				*p*
	*n* = 2554					*n* = 3136					*n* = 5690				
	Q1	Q2	Q3	Q4		Q1	Q2	Q3	Q4		Q1	Q2	Q3	Q4	
	*n* = 638	*n* = 639	*n* = 638	*n* = 639		*n* = 784	*n* = 784	*n* = 784	*n* = 784		*n* = 1423	*n* = 1422	*n* = 1423	*n* = 1422	
	**DTAC (mmol/d)**														
Mean ± SD	5.22 ± 1.75	9.44 ± 1.02	13.17 ± 1.20	21.67 ± 8.52	<0.0001	5.52 + 1.77	9.72 ± 1.00	13.14 ± 1.11	20.68 ± 9.07	<0.0001	5.38 ± 1.77	9.59 ± 1.01	13.15 ± 1.15	21.13 ± 8.84	<0.0001
Me, IQR	5.50	9.43	13.14	19.10		5.78	9.77	13.08	18.50		5.67	9.62	13.11	18.73	
	(3.99–6.67)	(8.52–10.34)	(12.12–14.18)	(16.90–23.03)		(4.37–7.08)	(8.85–10.61)	(12.15–14.11)	(16.52–21.56)		(4.18–6.87)	(8.71–10.47)	(12.13–14.16)	(16.76–22.20)	
Range	0.47–7.70	7.71–11.16	11.16–15.38	15.39–95.69		0.32–7.91	7.94–11.35	11.35–15.19	15.19–191.82		0.32–7.85	7.85–11.31	11.31–15.27	15.27–191.82	
	**DTAC/1000 kcal**														
Mean ± SD	3.15 ± 1.78	4.99 ± 2.07	6.15 ± 2.46	8.66 ± 4.34	<0.0001	4.55 ± 2.31	6.80 ± 2.69	8.50 ± 3.10	11.62 ± 7.16	<0.0001	3.88 ± 2.16	6.02 ± 2.59	7.48 ± 3.06	10.27 ± 6.24	<0.0001
Me, IQR	2.83	4.65	5.74	7.80		4.09	6.19	7.92	10.07		3.50	5.51	6.99	8.97	
	(2.02–3.90)	(3.65–5.84)	(4.55–7.21)	(5.89–9.98)		(3.03–5.55)	(4.98–7.99)	(6.43–10.02)	(8.16–13.05)		(2.46–4.83)	(4.26–7.14)	(5.42–8.92)	(6.89–11.65)	
Range	0.39–21.18	1.51–21.98	2.25–26.35	2.30–33.51		0.38–19.06	2.05–22.01	2.90–29.93	3.31–105.64		0.38–21.18	1.51–22.01	2.25–29.93	2.30–105.64	
	**Mean ^1^ (95% CI)**														
DTPI [mg/d]	1135	1738	2226	3187	<0.0001	1107	1714	2165	2962	<0.0001	1125	1729	2199	3067	<0.0001
	(1091–1179)	(1694–1782)	(2182–2270)	(3143–3231)		(1071–1143)	(1678–1750)	(2129–2200)	(2927–2998)		(1097–1153)	(1701–1757)	(2171–2227)	(3039–3095)	
DTPI/1000 kcal	654	886	1019	1251	<0.0001	876	1168	1372	1640	<0.0001	761	1028	1197	1450	<0.0001
	(626–682)	(858–914)	(991–1047)	(1223–1279)		(839–913)	(1131–1205)	(1335–1409)	(1603–1677)		(737–785)	(1004–1052)	(1173–1221)	(1426–1474)	
Vitamin C [mg/d]	60.1	79.3	92.8	117.7	<0.0001	67.2	85.7	94.8	133.7	<0.0001	64.2	82.1	93.5	125.8	<0.0001
	(54.0–66.2)	(73.2–85.4)	(86.7–98.9)	(111.6–123.8)		(60.5–74.0)	(79.0–92.4)	(88.0–101.5)	(126.9–140.4)		(59.6–68.8)	(77.5–86.7)	(88.9–98.1)	(121.2–130.4)	
Vitamin C/1000 kcal	34.9	40.6	42.1	46.5	<0.0001	54.4	57.9	60.2	74.0	<0.0001	44.9	49.1	50.9	60.5	<0.0001
	(31.7–38.1)	(37.4–43.8)	(38.9–45.3)	(43.3–49.7)		(49.3–59.5)	(52.9–63.0)	(55.1–65.3)	(69.0–79.1)		(41.8–48.1)	(45.9–52.2)	(47.7–54.0)	(57.4–63.7)	
Vitamin E [mg/d]	10.11	11.63	12.86	15.84	<0.0001	8.62	10.43	11.42	13.45	<0.0001	9.43	11.03	12.10	14.62	<0.0001
	(9.49–10.73)	(11.02–12.25)	(12.24–13.48)	(15.22–16.45)		(7.62–9.63)	(9.42–11.44)	(10.41–12.42)	(12.44–14.46)		(8.81–10.05)	(10.41–11.66)	(11.48–12.72)	(13.99–15.24)	
Vitamin E/1000 kcal	5.44	5.54	5.44	5.80	0.1965	6.44	6.81	7.10	7.05	0.6479	5.95	6.17	6.26	6.43	0.5209
	(5.17–5.70)	(5.27–5.80)	(5.18–5.71)	(5.53–6.06)		(5.66–7.23)	(6.03–7.60)	(6.31–7.88)	(6.26–7.84)		(5.50–6.40)	(5.72–6.62)	(5.81–6.71)	(5.98–6.88)	
β-Carotene [μg/d]	2313	2971	3194	3416	<0.0001	2598	2871	3086	3373	<0.0001	2488	2894	3142	3387	<0.0001
	(2075–2552)	(2733–3210)	(2955–3432)	(3178–3655)		(2364–2832)	(2638–3105)	(2853–3320)	(3139–3607)		(2320–2655)	(2727–3062)	(2974–3310)	(3219–3555)	
β-Carotene/1000 kcal	1428	1546	1432	1334	0.1568	2164	1967	1907	1841	0.0574	1812	1745	1666	1586	0.0309
	(1299–1558)	(1417–1676)	(1303–1562)	(1205–1463)		(1992–2337)	(1794–2139)	(1735–2080)	(1668–2013)		(1701–1924)	(1634–1857)	(1555–1778)	(1475–1698)	
Zinc [mg/d]	9.96	10.99	11.94	13.38	<0.0001	7.36	8.11	8.75	10.04	<0.0001	8.71	9.52	10.34	11.68	<0.0001
	(9.58–10.34)	(10.61–11.37)	(11.56–12.32)	(13.00–13.76)		(7.07–7.65)	(7.82–8.40)	(8.46–9.04)	(9.75–10.33)		(8.48–8.94)	(9.29–9.76)	(10.11–10.58)	(11.45–11.92)	
Zinc/1000 kcal	5.35	5.20	5.07	4.93	<0.0001	5.51	5.18	5.26	5.28	0.0459	5.43	5.19	5.16	5.12	0.0004
	(5.22–5.48)	(5.07–5.33)	(4.94–5.20)	(4.80–5.06)		(5.34–5.68)	(5.01–5.35)	(5.09–5.43)	(5.11–5.45)		(5.32–5.54)	(5.08–5.30)	(5.05–5.28)	(5.00–5.23)	
Iron [mg/d]	10.26	11.58	13.29	15.12	<0.0001	8.50	9.42	10.65	12.79	<0.0001	9.39	10.50	11.91	13.99	<0.0001
	(9.81–10.72)	(11.13–12.04)	(12.83–13.74)	(14.67–15.58)		(7.94–9.05)	(8.86–9.97)	(10.10–11.20)	(12.24–13.34)		(9.03–9.76)	(10.14–10.87)	(11.54–12.28)	(13.62–14.35)	
Iron/1000 kcal	5.52	5.56	5.68	5.62	0.5867	6.30	6.07	6.48	6.70	0.0567	5.89	5.82	6.06	6.19	0.0411
	(5.35–5.69)	(5.39–5.73)	(5.51–5.85)	(5.45–5.79)		(5.97–6.63)	(5.75–6.40)	(6.15–6.80)	(6.37–7.03)		(5.69–6.09)	(5.63–6.02)	(5.86–6.26)	(5.99–6.39)	
Manganese [mg/d]	3.67	4.40	4.85	5.80	<0.0001	3.18	3.79	4.21	4.92	<0.0001	3.43	4.09	4.54	5.36	<0.0001
	(3.50–3.83)	(4.24–4.57)	(4.69–5.02)	(5.63–5.96)		(3.05–3.31)	(3.66–3.92)	(4.08–4.34)	(4.79–5.05)		(3.33–3.53)	(3.99–4.20)	(4.43–4.64)	(5.25–5.46)	
Manganese/1000 kcal	2.10	2.19	2.17	2.23	0.1706	2.50	2.55	2.62	2.65	0.1216	2.29	2.37	2.39	2.45	0.0067
	(2.02–2.18)	(2.10–2.27)	(2.09–2.25)	(2.15–2.31)		(2.41–2.60)	(2.46–2.65)	(2.52–2.71)	(2.56–2.75)		(2.23–2.36)	(2.31–2.44)	(2.33–2.46)	(2.39–2.51)	
Copper [mg/d]	0.96	1.13	1.32	1.61	<0.0001	0.79	0.96	1.10	1.39	<0.0001	0.87	1.05	1.21	1.50	<0.0001
	(0.92–0.99)	(1.09–1.17)	(1.28–1.36)	(1.58–1.65)		(0.75–0.82)	(0.93–1.00)	(1.07–1.13)	(1.36–1.43)		(0.85–0.90)	(1.02–1.07)	(1.18–1.23)	(1.48–1.53)	
Copper/1000 kcal	0.523	0.548	0.575	0.607	<0.0001	0.594	0.626	0.674	0.746	<0.0001	0.557	0.587	0.624	0.679	<0.0001
	(0.507–0.538)	(0.532–0.563)	(0.560–0.591)	(0.592–0.623)		(0.573–0.615)	(0.605–0.647)	(0.653–0.695)	(0.725–0.767)		(0.543–0.570)	(0.573–0.600)	(0.611–0.638)	(0.666–0.693)	

^1^—Adjusted for age in men and women and for sex and age in the total group; *n*—number; DTAC—dietary total antioxidant capacity; DTPI—dietary total polyphenol intake; Me—median; IQR—interquartile range; Q1–Q4—quartiles of DTAC; SD—standard deviation. Differences in DTAC and DTAC adjusted for energy were tested by nonparametric Kruskal–Wallis test, while other variables were compared using a general linear model.

**Table 4:** Prevalence and OR (95% CI) of CVDs by quartiles of DTAC according to sex and overall.

Variables	Men				Women				Total			
	*n* = 2554				*n* = 3136				*n* = 5690			
	Q1	Q2	Q3	Q4	Q1	Q2	Q3	Q4	Q1	Q2	Q3	Q4
	*n* = 638	*n* = 639	*n* = 638	*n* = 639	*n* = 784	*n* = 784	*n* = 784	*n* = 784	*n* = 1423	*n* = 1422	*n* = 1423	*n* = 1422
CVD Prevalence %	23.1	21.8	17.2	15.2	23.7	21.1	18.3	19.0	23.3	21.4	18.1	17.2
(95% CI) ^A^	(20.4–25.9)	(19.0–24.6)	(14.4–20.0)	(12.4–18.0)	(21.1–26.3)	(18.5–23.8)	(15.7–21.0)	(16.4–21.6)	(21.4–25.2)	(19.5–23.4)	(16.2–20.0)	(15.3–19.1)
*p* *	-	-	-	0.0001	-	-	-	0.0205	-	-	-	<0.0001
OR	1	0.921	0.643	0.563	1	0.796	0.650	0.617	1	0.852	0.666	0.593
(95% CI) ^1^	-	(0.710–1.196)	(0.487–0.848)	(0.424–0.749)	-	(0.629–1.006)	(0.510–0.829)	(0.483–0.788)	-	(0.716–1.015)	(0.555–0.799)	(0.492–0.714)
*p*	-	0.5387	0.0018	<0.0001	-	0.0562	0.0005	0.0001	-	0.0733	<0.0001	<0.0001
AOR	1	0.934	0.706	0.631	1	0.884	0.731	0.880	1	0.906	0.733	0.770
(95% CI) ^2^	-	(0.685–1.274)	(0.511–0.976)	(0.452–0.882)	-	(0.675–1.157)	(0.554–0.965)	(0.663–1.167)	-	(0.740–1.111)	(0.595–0.904)	(0.621–0.955)
*p*	-	0.6654	0.0353	0.0071	-	0.3692	0.0268	0.3744	-	0.3432	0.0037	0.0176
AOR	1	0.916	0.698	0.610	1	0.882	0.726	0.877	1	0.904	0.726	0.752
(95% CI) ^3^	-	(0.671–1.251)	(0.504–0.967)	(0.436–0.855)	-	(0.673–1.155)	(0.550–0.958)	(0.660–1.165)	-	(0.737–1.108)	(0.588–0.895)	(0.605–0.935)
*p*	-	0.5830	0.0306	0.0041	-	0.3604	0.0237	0.3636	-	0.3304	0.0028	0.0102

*n*—Number; OR—odds ratio; AOR—adjusted odds ratio; CI—confidence interval; OR—odds ratio; CVD—cardiovascular disease; DTAC—dietary total antioxidant capacity; ^A^—adjusted for age in men and women and for sex and age in the total group; ^1^—crude OR in men and women or adjusted for sex in the total group; ^2^—adjusted for age, BMI, HDI, smoking status, and alcohol intake in men and women and additionally for sex in the total group; ^3^—adjusted for age, BMI, HDI, smoking status, alcohol intake, educational level, and physical activity in men and women and additionally for sex in the total group; *—*p*-value for comparisons between adjusted means. Difference in CVD prevalence over quartiles was tested using a general linear model, while logistic regression analysis was performed to estimate the odds of CVD between pairs of quartiles: Q2 vs. Q1, Q3 vs. Q1, and Q4 vs. Q1 for each model.

## Data Availability

All data in this study are available upon request to the authors at the following e-mail address: malgorzata.zujko@umb.edu.pl or awaskiewicz@ikard.pl.
